# Effects of combined exercises on shoulder mobility and strength of the upper extremities in breast cancer rehabilitation: a 3-week randomized controlled trial

**DOI:** 10.1007/s00520-023-07959-1

**Published:** 2023-09-01

**Authors:** Dominique Michels, Stefan König, Alexandra Heckel

**Affiliations:** 1 Institute of Sports Science, University of Tuebingen, 72074 Tuebingen, Germany; 2 University of Education Weingarten, 88250 Weingarten, Germany

**Keywords:** Breast cancer rehabilitation, Physical activity, Exercise therapy, Strength training, Mobility training

## Abstract

**Purpose:**

The aim of this study was to analyze the effects of mobility training with FIVE® devices in combination with device-supported strength exercises for shoulder mobility and strength of the upper extremities in women with breast cancer.

**Methods:**

We conducted a pretest-posttest intervention study with female breast cancer patients (*n* = 41) who were randomly assigned to two groups by lot during their stationary follow-up treatment at a rehabilitation clinic in the south of Germany between February and March 2020. As part of exercise therapy, the intervention group (*n* = 24) performed a mobility training with FIVE® devices combined with device-supported strength training, whereas the control group (*n* = 17) completed device-supported strength training. Before and after the 3-week intervention (3 training sessions/week), shoulder mobility and isokinetic maximal strength were tested.

**Results:**

Both groups achieved significant improvements in shoulder mobility in the frontal and sagittal plane (between 3.8 and 15.35%; *p* < 0.05) and in strength performance (31.36% [IG] vs. 51.24% [CG]; *p <* 0.001). However, no robust evidence could be determined about potential interaction effects.

**Conclusion:**

A combined device-supported strength and mobility training (FIVE®) showed no advantages. Therefore, a variety of exercise methods is possible in exercise therapy of breast cancer patients.

**Clinical trial registration number:**

Since the University of Education Weingarten does not assign clinical trial registration numbers or ethical approval numbers, none could be assigned for this study.

**Supplementary Information:**

The online version contains supplementary material available at 10.1007/s00520-023-07959-1.

## Introduction

Breast cancer is the most common cancer worldwide and a leading cause of death in women [1]. Breast cancer mortality rates have increased significantly worldwide over the past 25 years, which could be due to the increasing prevalence and growth of new cases of this type of cancer [2]. This trend should be one more reason to reinforce research in the field of prevention, treatment, and rehabilitation of breast cancer patients.

Over the last two decades, numerous studies have been conducted to examine the association between physical activity (PA) and breast cancer risk. There is consistent evidence that physical activity reduces breast cancer risk and the risk of recurrence. Moreover, several studies confirm the association of physical activity and a lower risk of death [3–5].

In addition to primary prevention and prevention of breast cancer recurrence, it has also been found out that PA has positive effects on treatment- and disease-related symptoms. Breast cancer patients frequently report side effects such as decreased physical performance and physical functioning (e.g., reduced upper extremity strength and mobility), as well as fatigue, polyneuropathy, and lymphedema during and after breast cancer treatment. Regarding lymphedema, it was shown that the volume of the arm in breast cancer patients can be reduced by weight lifting or resistance exercise [6]. These findings suggest that resistance training may be beneficial in patients with breast-cancer-related lymphedema or patients at risk of developing lymphedema. Further studies show that resistance exercises have a positive effect on fatigue [7, 8]. Comparable results can be achieved through combined aerobic and resistance exercises [9, 10]. Current studies examine whether exercise training influences chemotherapy-induced peripheral neuropathy (CIPN) [11, 12]. However, at present, there is still insufficient evidence of research regarding exercise training in breast cancer patients and CIPN.

In addition, various studies have described the improvement or, at least, the preservation of breast cancer patients’ physical performance by resistance and aerobic exercise interventions. In this context, several relevant findings have to be mentioned: First, studies having implemented resistance exercise or combined resistance and aerobic training showed the greatest impact on muscular strength [9]. Second, improvements in cardiovascular fitness have been observed in studies with aerobic exercises or combined resistance and aerobic training [9, 13]. Finally, there is evidence that breast cancer patients benefit from a combined aerobic and resistance exercise training regarding active range of motion of the shoulder and upper extremity isometric strength [14]. To sum up, we can assume that physical fitness and physical functionality can be regained in breast cancer patients after treatment.

Nevertheless, the impact of mobility exercises in combination with resistance training on physical function of the upper extremities has not been adequately analyzed so far. Considering the most frequent side effect of breast cancer treatment, i.e., a restricted shoulder mobility and reduced strength of the upper extremities, we can suppose that breast cancer patients would particularly benefit from a combined strength and mobility training. As a consequence, there is a need for research to examine the effectiveness of a combined mobility and strength training in breast cancer rehabilitation.

Therefore, the aim of this article is to present a study on the effects of combined strength and mobility training on shoulder mobility and strength performance of the upper extremities in breast cancer patients. We wanted to address the following research questions: (1) Which intervention effects on shoulder mobility and (2) which intervention effects on strength of the upper extremities (the right and left shoulder and shoulder girdle) can be ascertained and measured? As to both our theoretical analyses and the state of the art we hypothesized an increase of mobility and strength in the upper extremities of treated breast cancer patients during their rehabilitation.

## Methods

The 3-week randomized controlled trial (RCT) was conducted as a pre- and posttest intervention study at a rehabilitation clinic in Bad Waldsee between February and March 2020. Beforehand, we had received ethical clearance from the University of Education Weingarten.

### Sample size calculation and eligibility criteria

Initially, we computed the necessary sample size with an a priori power analysis using G*Power 3.1.9.4. For this purpose, we used empirical values from a former study as well as theoretical and methodological considerations (α = .05, 1-β = .80, f = 0.20) [12]. Our calculations revealed a necessary sample size of *n* = 52. Eligibility criteria included confirmed invasive breast carcinoma, completed adjuvant therapy except hormone therapy as well as the absence of chronic and orthopedic conditions that would preclude participation (e.g., severe heart failure). Patients with bone metastases, breast implants less than 1 year old, fever or current infectious diseases, and pregnant patients had to be excluded. This restriction was due to the bench press isokinetic maximum strength test, which imposes a significant physical strain on the test participants.

### Allocation and group distribution

Interested and eligible patients were informed about the procedure, and possible contraindications were explained and clarified by the study director. Participation in the study required a physician’s approval. All participants without contraindications received a declaration of consent. Voluntary consent by signature was required for participation in the study. Furthermore, we intended to allocate our participants to either the intervention or the control group by drawing lots at random.

In reality, the following situations occurred:

- In the beginning of the study, we had nearly enough participants available (*n* = 51) who could also be assigned in equal shares to the two groups.
- Due to corona pandemic, the study had to be terminated prematurely, which explains the small sample size (*n* = 41) and the non-existent 1:1 distribution of the intervention and control group (8 dropouts in the control group, 2 in the intervention group). Finally, the intervention group (IG) consisted of 24 and the control group (CG) of 17 participants.

**Fig. 1:**
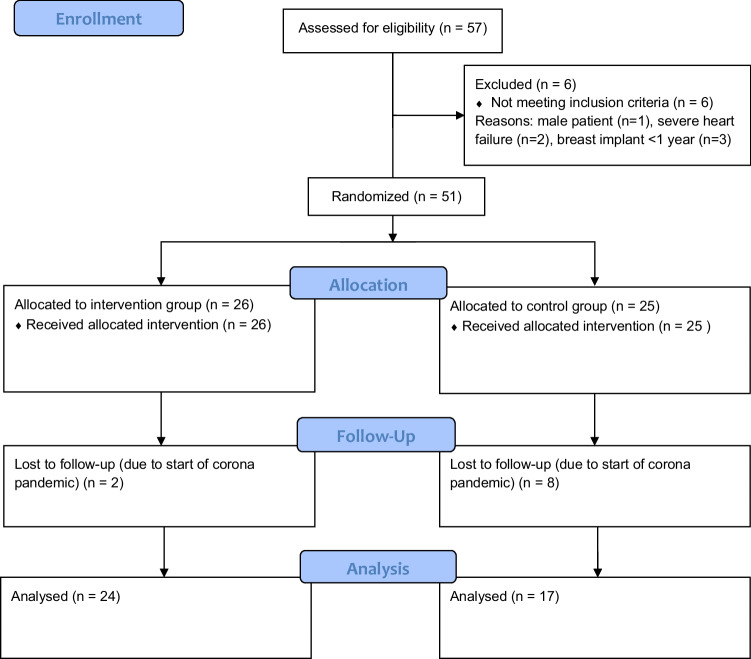
CONSORT flow diagram

In the end, a total of 41 female breast cancer patients (age: 56.68 ± 9.87 years; height: 164.93 ± 6.94 cm; weight: 76.61 ± 15.10 kg; BMI: 28.18 ± 5.33) took part in this study. As part of an adjuvant therapy, 51.2% of our participants had received chemotherapy (IG: *n* = 11, CG: *n* = 10), whereas 90.2% had undergone radiation therapy (IG: *n* = 16; CG: *n* = 21); consequently, there have been participants who have received both chemotherapy and radiation. The CONSORT flow diagram (Fig. 1) shows the different phases of the randomized trial of the two groups.

### Intervention programs and exercise details

The intervention program for both groups was a supervised multi-modal workout. The exercise plan for both groups is provided as supplementary material (Online Resource 1). Each training session lasted about 60 min and was performed as part of medical training therapy in an inpatient rehabilitation clinic. Training took place three times per week with approximately 48 h of rest between sessions. After the warm-up (bicycle ergometer or cross trainer, 10 min), both groups completed a device-supported full-body strength training.

The strength exercises were divided into two categories: upper extremity (UE) and lower extremity (LE). To avoid under- or overstrain, the participants were required to perform a minimum of three and a maximum of five exercises of each category. The strength exercises were:

- UE: seated rowing, chest press, butterfly reverse, lat. pulldown, straight arm-pulldown
- LE: leg extension, back extension, abductor, leg curls, leg press

For each exercise, two sets of 15 repetitions were trained with a 30-s rest between sets. Exercise intensity was determined by the patient’s ability. The goal was to achieve a moderate to vigorous level, corresponding to perceived exertion of 13–15 on the Borg scale. Progression was achieved by increasing the initial weight. All participants were instructed to gradually increase the weight by a maximum of one level per session. All strength exercises were performed on MILON® Premium Med devices, with one exception: straight arm-pulldowns were conducted using a cable machine. Unlike conventional devices, Milon® devices are electronically controlled and require an additional retraction weight to be moved during the eccentric movement. Due to chip card control, all Milon® devices adjust individually to the user after initialization (ergonomic and weight settings). The chip card was also used to record training progression and document the devices used in every session.

In addition, a balance board (Posturomed®) was used for exercises as part of the training. The participants had to keep their balance for 10–20 s while standing on one leg. The device can be set to three levels of difficulty (1–3), where 1 means “rather easy” and 3 means “rather difficult.” This exercise had to be performed twice to three times with each leg. At the end of each training session, all participants had to use a ball (Blackroll®), Duoball (Blackroll®) or fascia stick to stimulate the muscles and fascia of the shoulder girdle. Some studies confirm the positive correlation between the use of muscle rollers and improved body mobility and increased range of motion [15].

Participants of the intervention group also completed mobility training, which comprised mobility exercises for the torso and shoulder girdle on five different devices (FIVE ®). They are provided as supplementary material (Online Resource 2). Participants were instructed to perform three sets of each mobility exercise, staying in the maximum range of motion for 20 s with 20–30 s of rest in between. Individual exercise intensity was adjusted during each mobility session. Mobility training was performed after strength training (3×/week) and lasted approximately 15–20 min.

All exercises were performed individually and supervised by at least one qualified sport therapist who provided corrections and instructions. Adherence to exercise was monitored and documented by the supervising sport therapist. There were no adverse events reported during the intervention period, except for a general state of fatigue in both groups and stretching pain during the flexibility exercises in the intervention group. Subjects in both groups were requested to follow only the exercises assigned to their group and to not make any changes in their exercise behavior during the study period. However, in accordance with a holistic approach to therapy, the participants also attended further complementary therapy programs (pilates, water aerobics, disease-specific group training, Nordic walking) and counseling services (return-to-work and nutritional counseling).

### Data collection and analysis

Data was collected through individual motor tests before and after intervention (3 weeks). In this process, shoulder mobility was measured on both sides using a laser device, in both sagittal and frontal plane (lateral arm movement and forward flexion). Shoulder height and arm length were quantified (in neutral subtalar position), as was the diagonal from the fingertips to the floor at maximum abduction. The inner shoulder angle was described using the law of cosine. Strength performance was tested by an electronically operated bench press (Milon®). The test consisted of three attempts, so the measured force corresponded to approximately 93% of the maximum force (1RM). The lowest recommended weight (in kg) was noted for further analysis. To ensure equal conditions for each participant, all measurements were carried out by the study director and in the same chronological order. After data collection and preparation, data was tested for normal distribution using the Kolmogorov-Smirnow test and histograms; all data met this criterion.

Data analysis was realized using descriptive statistics as well as individual graphical growth plots to provide transparency of raw data and avoid the superficiality of average growth trajectories [16]; in addition, we plotted 95% confidence intervals to have robust measures of dispersion and to illustrate information of significance testing [17]. To test main and interaction effects for statistical significance, we conducted repeated measures ANOVA (2 × 2) and enhanced these findings with effect size ω^2^ due to our small sample size [18]. Finally, *t*-tests for matched pairs were computed including effect sizes Hedges’ g to compare and discuss the practical significance [19] of the two programs.

## Results

Tables 1 and 2 present the anthropometric data and descriptive coefficients of our most relevant dependent variables. Additionally, we have added diagrams visualizing the raw data distribution in our supplementary materials (Online Resource 3) to show inter-individual variation [19, 20].

**Table 1:** Antropometric data (mean, 95% CI) and received type of therapy (*n* = 41)

Parameter	Group_1 [IG], *n* = 24	Group_2 [CG], *n* = 17
Age	52.38 (48.78–55.97)	62.76 (58.40–67.13)
Height (in cm)	165.08 (162.33–167.84)	164.71 (160.76–168.65)
Weight (in kg)	77.21 (71.85–82.57)	75.76 (66.32–85.21)
BMI	28.43 (26.34–30.52)	27.83 (24.83–30.89)
Chemotherapy	11	10
Radiotherapy	21	16

**Table 2:** Results (mean, SD) of motor tests

Test item		Group_1 [IG], *n* = 24		Group_2 [CG], *n* = 17	
		Pretest	Posttest	Pretest	Posttest
Mobility frontal (in °)	Right	118.67 (28.88)	136.88 (25.20)	128.41 (23.91)	137.41 (17.26)
	Left	126.29 (29.44)	137.54 (23.89)	115.88 (29.57)	127.06 (37.62)
Mobility sagittal (in °)	Right	141.96 (15.03)	148.50 (18.46)	147.12 (18.78)	153.12 (19.10)
	Left	143.58 (24.89)	149.04 (19.31)	136.47 (24.94)	144.35 (23.67)
Strength (in kg)		36.63 (15.97)	48.12 (19.77)	24.04 (12.65)	36.36 (17.05)

Normal range of motion (ROM) of the shoulder joint has been specified by Schünke to be 180° in frontal plane and 170° in sagittal plane [21]. Therefore, the participants have approximately 70% of normal shoulder ROM in frontal plane and approximately 85% in sagittal plane at baseline. Regarding the percentage improvement of ROM, it has been shown that the intervention group, as well as the control group achieved significant improvements in shoulder mobility in frontal plane (right: 15.35% vs. 7%, *p <* 0.001; left: 8.91% vs. 9.65%, *p =* 0.004) and sagittal plane (right: 4.61% vs. 4.08%, *p =* 0.03; left: 3.8% vs. 5.77%, *p =* 0.049). The strength values improved from pre- to posttest by 31.36% in IG, whereas the CG achieved an improvement of 51.24% (*p <* 0.001). This large increase will be considered in the “Discussion” section.

Testing the baseline level of all dependent variables for statistically significant differences, *t*-tests brought to light that apart from variable “strength” (*t* = − 2,702, *df* = 39, *p* = .010) the dependent variables do not differ statistically. Consequently, we could assume that the participants started from comparable levels of frontal and sagittal mobility. The difference in strength must be taken into account in the “Discussion” section.

**Fig. 2:**
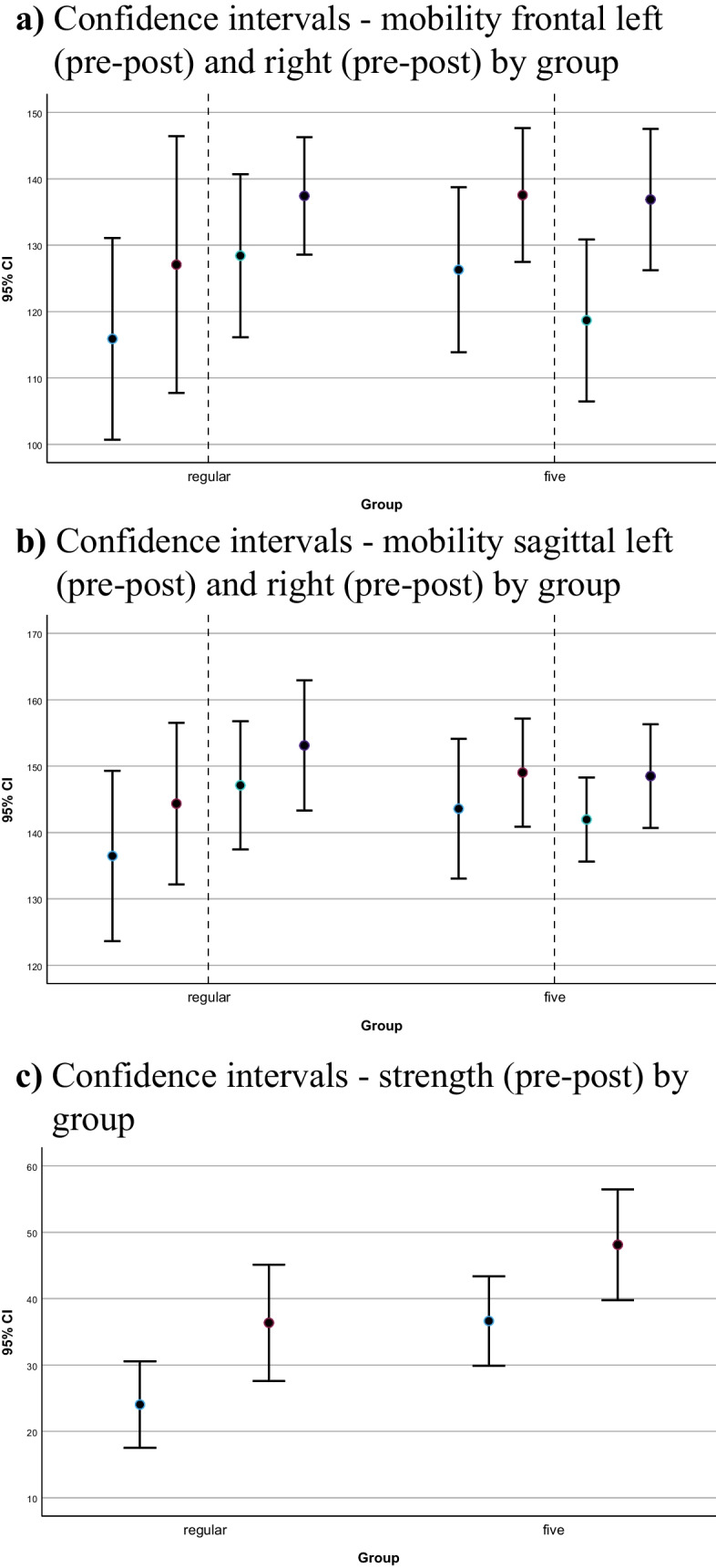
**a** Confidence intervals—mobility frontal left (pre-post) and right (pre-post) by group. **b** Confidence intervals—mobility sagittal left (pre-post) and right (pre-post) by group. **c** Confidence intervals—strength (pre-post) by group

Literature on null hypothesis testing as well as on longitudinal data analysis recommends an initial examination of empirical growth plots before computing inferential statistics. This corresponds with a first exploratory impression of change over time and has been conducted with confidence intervals (95%) for each dependent variable [16, 22]. They are presented in Fig. 2a–c.

The diagrams show changes in strength as well as in ROM in frontal and sagittal plane in both groups. The 95% confidence intervals indicate improvements for both groups from pre- to posttest in all test items.

An inferential analysis confirmed these results. For testing the exercise effects on all dependent variables for statistical significance, we conducted five 2 × 2 rANOVA (two groups, two measurement points); the results show main effects for all analyses with remarkable effect sizes, but no interaction effects at all [23]. All relevant coefficients are displayed in Table 3.

**Table 3:** Results of 2 × 2 rANOVA

	Main effect “time”			Interaction effect “time*group”		
	*F*	*p*	*ω*^2^	*F*	*p*	*ω*^2^
Mobility fr	16.374	.000	.278	1.876	.179	.021
Mobility fl	9.335	.004	.172	.000	.992	.000
Mobility sr	5.053	.030	.092	.009	.923	.000
Mobility sl	4.146	.049	.080	.137	.713	.000
Strength	21.897	.000	.343	.027	.871	.000

Abbreviation: fr = frontal right, fl = frontal left, sr = sagittal right, sl = sagittal left; ω^2^ = omega-square represented by 0.01 for small, 0.06 for moderate, and 0.14 for large effects [23]

It has to be reported that both groups (main effect) show statistically significant improvements with effect sizes (omega^2^) between 8 and 34%, i.e., with a middle to a very high explanation of variance [23]; in contrast, no group effect (interaction) could be ascertained.

To control for the potential influence of the type of oncological treatment, the between-subject factors “chemotherapy” and “irradiation” were included as part of an additional statistical analysis. It can be observed that the between-subject factor “chemotherapy” has no statistically significant influence on the dependent variables (mobility and upper extremity strength), while the between-subject factor “irradiation” shows a significant influence *F*(1,37) = 5.781, *p= .*021, ω^2^= .135 with a medium to large effect on mobility in the sagittal plane on the right side. Including further independent variables and covariates into the statistical models, e.g., level of pain, BMI, age, did not improve the fit indices of the analyses. Thus, we conclude that the simple main effect model explains our data best.

Finally, two further issues should be mentioned: First, the goal of improving one’s physical abilities usually depends on both, a certain exercise frequency and a total duration of the intervention. Whereas the second factor was limited to 3 weeks due to demands of health insurance companies, we assumed the total amount of training lessons to be an important covariate for effect of the intervention. In fact, there was a rather large range from 5 to 11 units (M = 8, SD: 1.643) which supported this assumption. However, none of our dependant variables (cf. Table 3) was influenced by this covariate, a fact that needs further discussion. Second, breast cancer patients usually suffer from various types of pain. For this reason, the participants of our study received a questionnaire on pain, which yielded the following results: approximately 88% of all breast cancer patients suffer from pain in everyday life. Most of the participants had scar pain owing to surgical treatment. About 15 out of 41 patients reported numbness in their hands or feet (polyneuropathy) or other kind of pain related to nerve problems. Therefore, it is reasonable to assume that restrictions in shoulder mobility or strength may also occur as a result of various types of pain, such as scar pain or polyneuropathy.^1^

To obtain a greater understanding of the effects of the two programmes, we finally conducted *t*-tests with matched pairs for each group and each dependant variable; to avoid an accumulation of α-error, we applied Bonferoni correction which provided α’ = .01 (α’ = .05/5 = .01). In addition, Hedges’ *g* was computed to provide a basis for an analysis of the practical significance of our intervention. Tables 4 and 5 give an overview of the findings for both groups.

**Table 4:** Results of *t*-tests with matched pairs for control group

Control group (regular exercise training, *n* = 17)					
Dependent variable	Time point	Mean	SD	*P*	*g* (95% CI)
Mobility frontal right (mfr)	Pre	128.41	23.906	.131	0.377 [0.110; 0.854]
	Post	137.41	17.256		
Mobility frontal left (mfl)	Pre	115.88	29.565	.149	0.359 [0.127; 0.834]
	Post	127.06	37.622		
Mobility sagittal right (msr)	Pre	147.12	18.781	.247	0.284 [0.194; 0.754]
	Post	153.12	19.101		
Mobility sagittal left (msl)	Pre	136.47	24.935	.099	0.414 [0.077; 0.894]
	Post	144.35	23.667		
Strength (stre)	Pre	24.04	12.648	.006	0.743 [0.205; 1.264]
	Post	36.36	17.048

**Table 5:** Results of *t*-tests with matched pairs for target group

Target group (exercise training + FIVE®, *n* = 24)					
Dependent variable	Time point	Mean	SD	*P*	*g* (95% CI)
Mobility frontal right (mfr)	Pre	118.67	28.882	.000	0.912 [0.432; 1,378]
	Post	136.88	25.199		
Mobility frontal left (mfl)	Pre	126.29	29.442	.000	0.679 [0.234; 1.113]
	Post	137.54	23.887		
Mobility sagittal right (msr)	Pre	141.96	15.026	.002	0.424 [0.008; 0.832]
	Post	148.50	18.463		
Mobility sagittal left (msl)	Pre	143.58	24.894	.008	0.244 [0.158; 0.641]
	Post	149.04	19.311		
Strength (stre)	Pre	36.63	15.974	.001	0.708 [0.259; 1.145]
	Post	48.12	19.767		

**Fig. 3:**
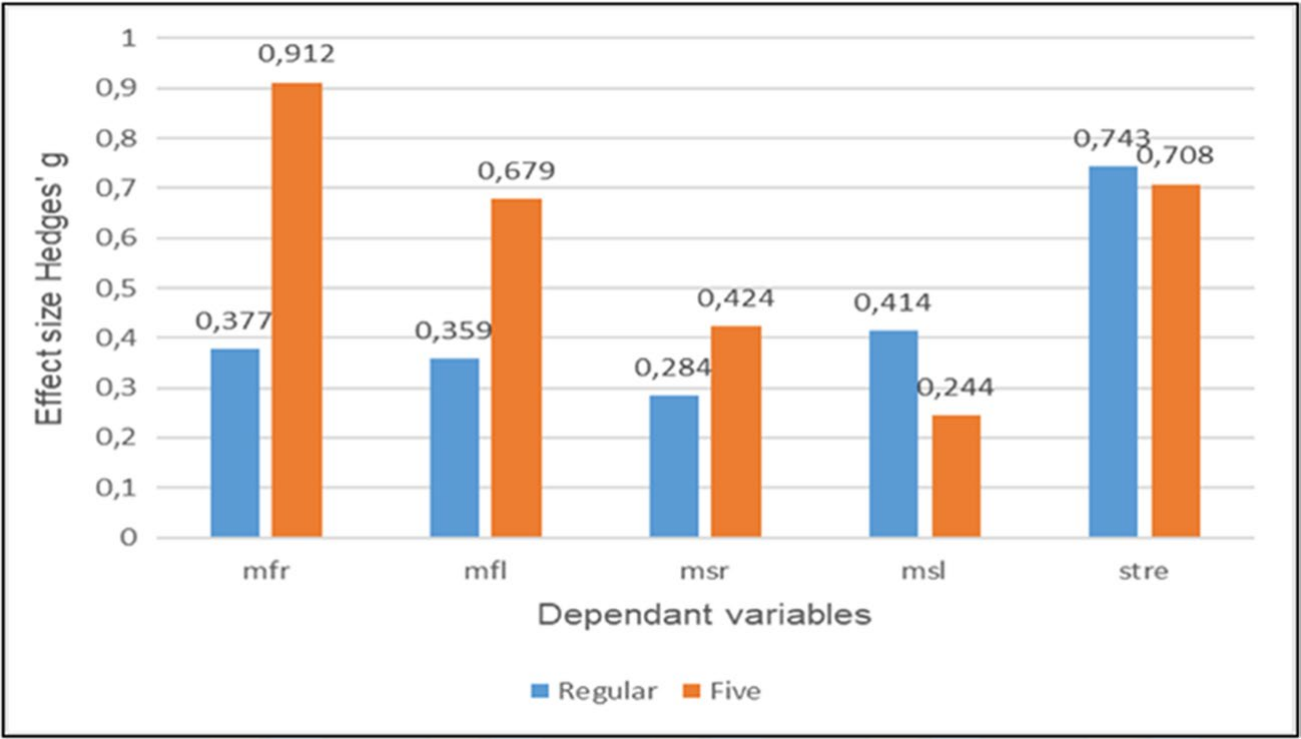
Comparison of effect sizes Hedges’ *g* (0.2 = small, 0.5 = moderate, 0.8 = large) [24]

The results show that our target group improved statistically significant in each item with small to moderate effects. In contrast, the control group could only advance strength with a moderate effect; all other effects were small. Finally, we compared the effect sizes graphically to provide a basis for analyzing and interpreting the practical significance of the intervention programs; the results are presented in Fig. 3. As we expected, the combined program seems to be more effective in terms of mobility, whereas both resulted in nearly equal enhancements in terms of strength.

## Discussion

The purpose of this study was to investigate the effects of combined exercises on shoulder mobility and strength of the upper extremities in breast cancer rehabilitation. To our knowledge, this is the first study showing significant improvement in these outcomes with combined device-supported strength and mobility training (FIVE®, MILON®) in a sample of female breast cancer patients shortly after treatment. Previous studies examined the effects of aerobic and resistance training in breast cancer patients, but did not analyze the results of a combined mobility and resistance training on devices in breast cancer patients in rehabilitation [25, 26]. Apart from that, the implementation of FIVE® devices in exercise therapy has not been investigated for patients in breast cancer rehabilitation yet [27].

As a first consequence, we can conclude that both methods show small to large (practical) exercise effects for patients in breast cancer rehabilitation in each dependent variable. In detail and with reference to our statistical analyses, we could observe that Bonferroni corrected t-tests for matched pairs revealed that only the intervention group achieved statistically significant improvements in shoulder mobility, although repeated measures ANOVA showed no interaction effects with respect to all dependent variables. Additionally, only the treatment type “irradiation” had a significant influence on the mobility in the sagittal plane on the right side. This may be due to the fact that the lymph nodes are also treated during irradiation, which increases the risk of lymphatic congestion (lymphedema) in the chest or arm and can severely limit the patient’s mobility [27, 28].

In contrast, both groups improved their strength performance after a three-week combined strength and mobility training on FIVE® devices, respectively device-supported training. Because of the specificity of the two approaches, these results are in accordance with our initial theoretical assumptions, and they support and complement previous results concerning aerobic capacity, lower and upper limb strength, and agility [26, 27, 29].

Nevertheless, reasons for the lack of interaction effects in all outcomes require closer consideration: First, the short duration of this intervention may have been insufficient to promote significant differences in training methods; as we know from training science, it takes at least 5 to 6 weeks for measurable training effects [30]. However, guidelines of health insurance companies determine a duration of 3 weeks for rehabilitation; hence, patients should continue their program at home to meet the criteria for exercise theory. Second, the small sample size may be cited as a limitation, although an a posteriori control with G*Power 3.1.9.4 did not confirm this assumption. In our view, this calls for the necessity of replication studies with a focus on longitudinal designs [26].

Another and third issue to be considered is the varying amount of training lessons, which do not affect the dependant variables. The fact that there is a training effect although some patients completed just five training lessons may be explained by their participation in further therapy programs (e.g., pilates, water aerobics, cancer-specific group training, Nordic walking). These interventions aimed at treating gynecologic patients with breast, ovarian, uterine, or cervical cancer.

It is important to note that these therapies were designed for all gynecologic patients with different emphases such as relaxation and functional exercise. However, they were not specifically planned to treat the side effects associated with breast cancer. Therefore, the potential impact of these therapies on the study results should be considered as rather small. For ethical reasons, patients had to be allowed to participate in further therapy programs in order to enable a plenary therapeutic success, which may have led to some bias. Statistical analysis is needed to examine whether there is a bias caused by participation in other therapy programs and how these variables might have influenced the therapy success. Due to the large variety of complementary therapy programs, the influence of individual variables could not be statistically determined in this study [31].

In the current study, baseline levels of the dependent variables did not differ except the variable strength. Thus, the irregularity of the variable strength may be explained by a highly diverse fitness level of the patients due to different exercise habits and treatment options.

The different treatment options may have influenced the mobility results in the pre- and postintervention assessments as well. Patients indicated different results of the left and right shoulder mobility at baseline and postintervention. This may be illustrated by the fact that either the left or the right side was treated. The abovementioned improvement of the outcome strength might be attributed to the inclusion of women within a short period after breast cancer treatment and the physically inactive nature of the participants upon enrolment. Previous studies utilizing a combined aerobic and resistance exercise intervention showed significant improvements in strength performance, yet to a greater degree than our results [9, 14].

There are a few limitations to this intervention that demand discussion. First, this study has a small sample size, which results in issues such as reduced statistical power to identify signficant difference in training methods. In addition, in the current study, there was an uneven group assignment as well as a short duration of intenvention, which may have affected the statistical results in terms of effect size. Moreover, adherence to the exercise program is challenging, thus the amount of training lessons varies greatly. Strengths of our study include the focus on device-supported mobility training, the randomized controlled trial design, and the use of laser technology to objectively measure ROM in the shoulder joint.

## Conclusion

In conclusion, this study shows that device-supported strength training, both in isolated form and in combination with mobility training, leads to improvements in strength and shoulder mobility in the rehabilitation of female breast cancer patients. However, a more precise consideration unveiled that the combined intervention lead to higher and more substantial effect sizes in three dependent variables. We conclude that the results of this RCT study suggest that future trials with breast cancer patients in rehabilitation should be of longer duration to promote long-term effects that may show any difference in training methods. A larger RCT examining this issue is also desirable.

## Supplementary information

ESM 1 (DOCX 16 kb)

ESM 2 (DOCX 646 kb)

ESM 3 (DOCX 727 kb)

## Data Availability

The dataset is available via the RADAR research data service.

## Footnotes

1 Less than 10% of the data per individual and in the entire data set was missing.
