# Supplementary material for: Effects of combined exercises on shoulder mobility and strength of the upper extremities in breast cancer rehabilitation: a 3-week randomized controlled trial
Source: Support Care Cancer. 2023 Sep 1;31(9):550. doi: 10.1007/s00520-023-07959-1 (PMC10474198; doi:10.1007/s00520-023-07959-1)
Supplement: Supplementary file 1 — (DOCX 16 kb) [file 520_2023_7959_MOESM1_ESM.docx]

**Supplemental material**

**Online Resource 1** Exercise plan for IG and CG

| **Intervention group (IG)** | **Control group (CG)** |
| --- | --- |
| **Warm-up** | |
| - Bicycle ergometer or - Cross trainer | |
| **Strength training (MILON® Premium Med devices)** | |
| - Upper extremity: seated rowing, chest press, butterfly reverse, lat. pulldown, straight arm-pulldown - Lower extremity: leg extension, back extension, abductor, leg curls, leg press   The participants were required to perform a minimum of three and a maximum of five exercises of each category (UE/LE). | |
| **Mobility training (FIVE® devices)** |  |
| - Chest Mover - Lateral Mover - Calf stone - Breast-shoulder girdle - Forearms/hands |  |
| **Additional exercises** | |
| - Balance board (Posturomed®) - Fascia and muscle stimulation (Blackroll®, Fascia Stick) | |

For detailed information about the mobility exercises see Online Ressource 2

Effects of combined exercises on shoulder mobility and strength of the upper extremities in breast cancer rehabilitation: a three-week randomized controlled trial

Supportive Care in Cancer

Michels D, Heckel A, König S

dominique.michels@student.uni-tuebingen.de
