# Supplementary material for: Effects of combined exercises on shoulder mobility and strength of the upper extremities in breast cancer rehabilitation: a 3-week randomized controlled trial
Source: Support Care Cancer. 2023 Sep 1;31(9):550. doi: 10.1007/s00520-023-07959-1 (PMC10474198; doi:10.1007/s00520-023-07959-1)
Supplement: Supplementary file 2 — (DOCX 646 kb) [file 520_2023_7959_MOESM2_ESM.docx]

**Supplemental material**

**Online Resource 2** Five device-supported mobility exercises (FIVE®)

| **Component** | **Addressed parts of the body** | **Demonstration** |
| --- | --- | --- |
| Exercise 1  Chest-Mover | Shoulder and breast muscles  Intercostal muscles  Abdominal muscles  Shoulder girdle | 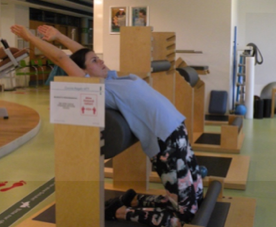 |
| Exercise 2  Lateral-Mover | Transverse abdominal muscles  Shoulder girdle muscles  Shoulder girdle | 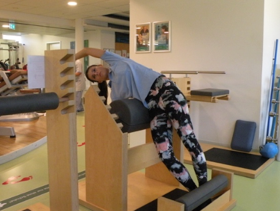 |
| Exercise 3  Calf stone | Hamstring muscles  Shoulder girdle muscles  Shoulder girdle | 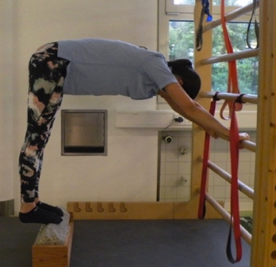 |
| Exercise 4  Breast-shoulder girdle | Shoulder and breast muscles  Shoulder girdle | 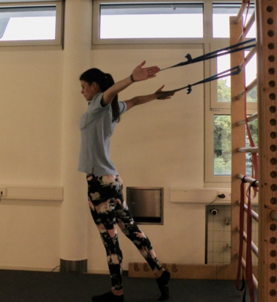 |
| Exercise 5 Forearms / hands | Forearm muscles  Wrist | 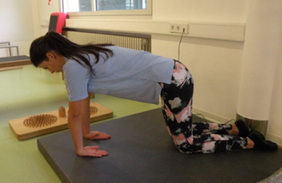 |
| Hold end position for 20 seconds or five deep breaths; 2-3 sets per exercise | | |

Effects of combined exercises on shoulder mobility and strength of the upper extremities in breast cancer rehabilitation: a three-week randomized controlled trial

Supportive Care in Cancer

Michels D, Heckel A, König S

dominique.michels@student.uni-tuebingen.de
