# Supplementary material for: Effects of combined exercises on shoulder mobility and strength of the upper extremities in breast cancer rehabilitation: a 3-week randomized controlled trial
Source: Support Care Cancer. 2023 Sep 1;31(9):550. doi: 10.1007/s00520-023-07959-1 (PMC10474198; doi:10.1007/s00520-023-07959-1)
Supplement: Supplementary file 3 — (DOCX 727 kb) [file 520_2023_7959_MOESM3_ESM.docx]

**Supplemental material**

**Online Resource 3** Raw data visualization

1. Mobility frontal right

**
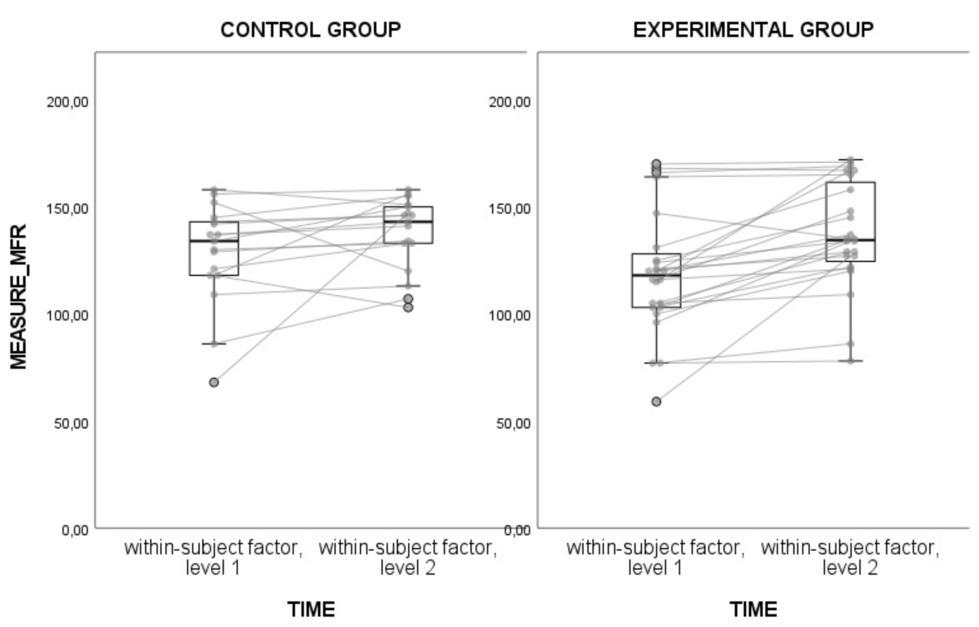
**

**b)** Mobility frontal left

**
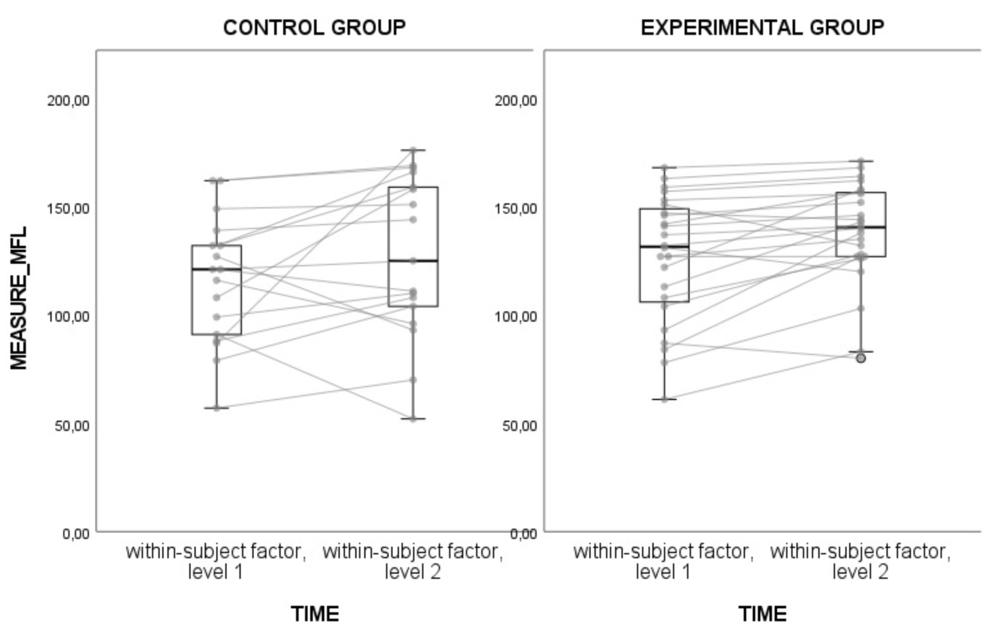
**

**c)** Mobility sagittal right

**
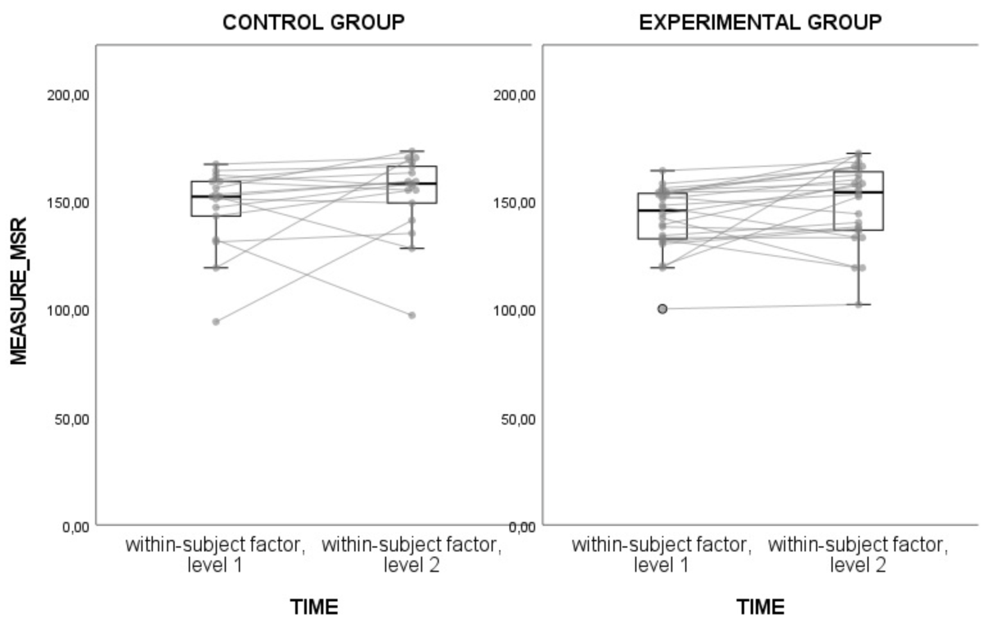
**

**d)** Mobility sagittal left

**
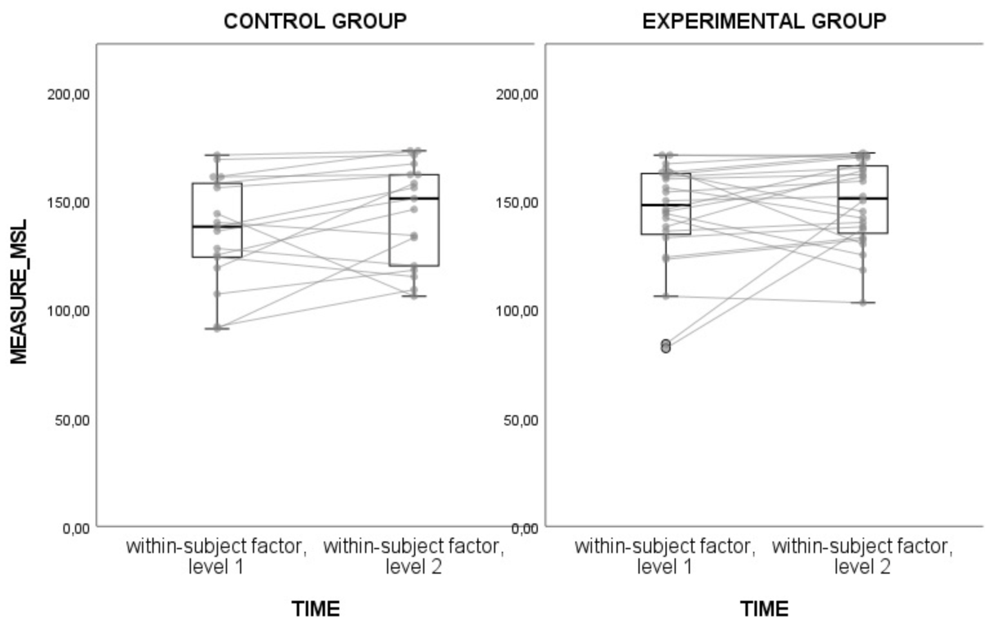
**

**e)** Strength


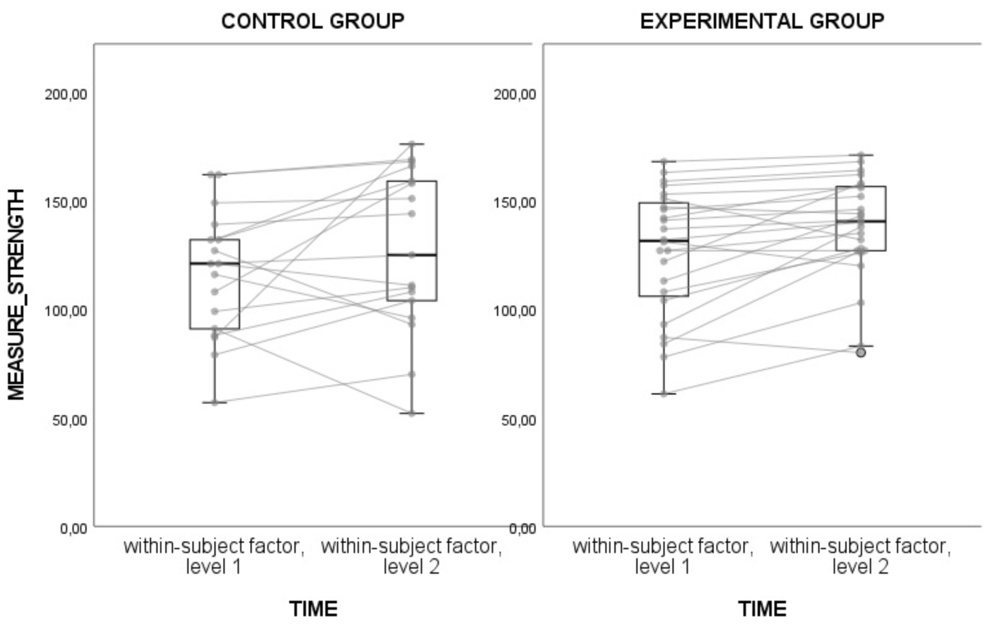


Effects of combined exercises on shoulder mobility and strength of the upper extremities in breast cancer rehabilitation: a three-week randomized controlled trial

Supportive Care in Cancer

Michels D, Heckel A, König S

dominique.michels@student.uni-tuebingen.de
